# The Impact of Extracellular Vesicle-Encapsulated Circulating MicroRNAs in Lung Cancer Research

**DOI:** 10.1155/2014/486413

**Published:** 2014-09-11

**Authors:** Yu Fujita, Kazuyoshi Kuwano, Takahiro Ochiya, Fumitaka Takeshita

**Affiliations:** ^1^Division of Molecular and Cellular Medicine, National Cancer Center Research Institute, 5-1-1 Tsukiji, Chuo-ku, Tokyo 104-0045, Japan; ^2^Division of Respiratory Diseases, Department of Internal Medicine, Jikei University School of Medicine, 3-19-18 Nishi-shinbashi, Minato-ku, Tokyo 105-8471, Japan

## Abstract

Lung cancer is the leading cause of cancer-related deaths. Biomarkers for lung cancer have raised great expectations in their clinical applications for early diagnosis, survival, and therapeutic responses. MicroRNAs (miRNAs), a family of short endogenous noncoding RNAs, play critical roles in cell growth, differentiation, and the development of various types of cancers. Current studies have shown that miRNAs are present in the extracellular spaces, packaged into various membrane-bound vesicles. Tumor-specific circulating miRNAs have been developed as early diagnostic biomarkers for lung cancer. Remarkably, some studies have succeeded in discovering circulating miRNAs with prognostic or predictive significance. Extracellular vesicles (EVs), such as exosomes and microvesicles, are recognized as novel tools for cell-cell communication and as biomarkers for various diseases. Their vesicle composition and miRNA content have the ability to transfer biological information to recipient cells and play an important role in cancer metastasis and prognosis. This review provides an in-depth summary of current findings on circulating miRNAs in lung cancer patients used as diagnostic biomarkers. We also discuss the role of EV miRNAs in cell-cell communication and explore the effectiveness of these contents as predictive biomarkers for cancer malignancy.

## 1. Introduction

Lung cancer remains the leading cause of cancer-related deaths in the world [1]. It is a heterogeneous disorder with two pathological types: non-small-cell lung cancer (NSCLC) and small-cell lung cancer (SCLC). Approximately 85% of all lung cancers are categorized as NSCLC. The most common pathological types of NSCLC are adenocarcinoma (30–50%) and squamous cell carcinoma (30%). Many therapeutic strategies, including surgery, radiotherapy, chemotherapy, and molecular target therapies, are commonly used to treat lung cancer, either alone or in combination. A majority of lung cancer patients are in advanced stages of the disease with limited treatment choices, mainly consisting of cytotoxic chemotherapeutic agents and targeted molecular therapies. Despite the development of novel targeted therapies, the prognosis for lung cancer remains poor due to drug resistance and tumor recurrence. Therefore, one of the major challenges in lung cancer research is the identification of stable biomarkers that can be routinely measured in samples that are accessible early. Biomarkers for lung cancer have raised great expectations in their clinical applications for early diagnosis, prognosis, and therapeutic responses. However, conventional serum markers, such as carcinoembryonic antigen (CEA) and squamous cell carcinoma antigen (SCC), lack sufficient sensitivity and specificity to facilitate early detection of lung cancer. Disease-driven proteomics based on mass spectrometry have serious technical limitations because of the complexity of the blood proteome. Recently, remarkable attention has been paid to cell-free nucleic acids, such as DNA, mRNA, and microRNA (miRNA), which are present at varying concentrations in the blood of cancer patients [2].

MiRNAs are endogenous, single-stranded, noncoding RNAs 19–22 nucleotides long that regulate translation through their interactions with mRNA transcripts [3]. MiRNAs are first transcribed, for the most part, by RNA polymerase II as a large primary miRNA (pri-miRNA) then processed by the endonuclease Drosha into a hairpin structure (precursor miRNA) and then further cleaved by the endonuclease Dicer into a single-stranded mature miRNA [4, 5]. The mature miRNA is incorporated into a complex known as the RNA-induced silencing complex (RISC), which contains the Argonaute 2 (AGO2) and glycine-tryptophan 182 kDa proteins (GW182). As a part of this complex, the mature miRNA regulates gene expression by binding to partially complementary sequences in the 3′-untranslated regions (3′UTRs) of the target mRNAs, leading to mRNA degradation or translation inhibition [6]. MiRNAs posttranscriptionally inhibit gene expression through multiple mechanisms, all of which involve base-specific interactions with target mRNA transcripts. A single miRNA may target dozens of mRNAs and one mRNA can be regulated by multiple miRNAs. In mammals, miRNAs are assumed to regulate more than 50% of all protein-coding genes [7]. This evidence indicates that miRNAs are not only important therapeutic targets but also promising biomarkers, as their expression could reflect the information dispersed on thousands of target genes [8]. Recently, dysregulation of miRNA expression was determined to contribute to various physiological and pathological processes, such as development, differentiation, cell proliferation, apoptosis, and homeostasis [9, 10]. A number of researchers have also demonstrated that aberrant miRNA expression is related to cancer development and progression, behaving as tumor suppressors or oncogenes [11]. Some miRNAs are overexpressed or downregulated in certain cancer types. Expression patterns of miRNAs are unique to individual tissue types and differ between cancer and normal samples [12, 13]. Current data support the potential of miRNAs as biomarkers for NSCLC [13–20]. Moreover, assessment of multiple miRNA expression levels can accurately predict prognosis and survival in lung cancer [21].

Over the last few years, recent studies have shown that miRNAs are present in extracellular spaces, such as blood, urine, and saliva [2]. MiRNAs can be secreted via extracellular vesicles (EVs) and by protein-miRNA complexes, such as high-density lipoprotein (HDL) and AGO2, which is part of the RISC [22, 23]. Circulating miRNAs are sensitive to protease treatment of plasma but are protected from plasma RNase digestion [24]. Resistance to the condition has been attributed to the above-described encapsulation and association with protein complexes [25, 26]. Circulating miRNAs are one of the most promising next-generation biomarkers for cancer diagnosis. Moreover, the involvement of EVs in cancer biology is now of great interest. Recent studies have shown that EVs serve as versatile intercellular communication vehicles [27]. These findings have established the novel concept that EV miRNAs have potential not only as putative biomarkers but also for reflecting cancer progression. Here, we provide a perspective on the potential contribution of circulating miRNA and EV research to the development of cancer diagnosis and therapeutics.

## 2. Mechanism of MicroRNA Release into Extracellular Spaces

**Figure 1:**
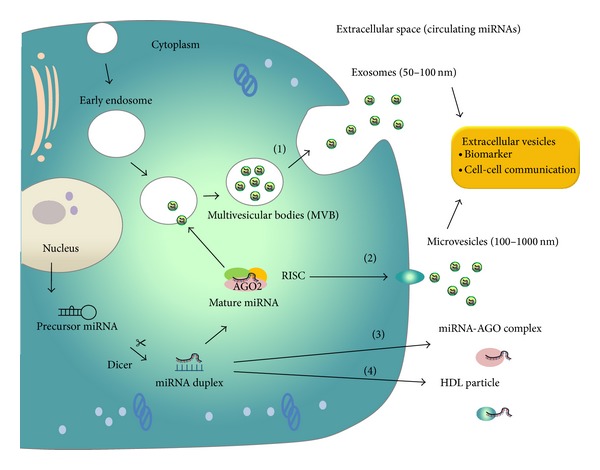
MiRNA release mechanisms into extracellular space. Precursor miRNAs are processed by ribonuclease Dicer to mature double-stranded miRNAs (miRNA duplex). One strand of the miRNA duplex is selectively loaded into the RNA-induced silencing complex (RISC), which contains the Argonaute (AGO) family protein as a core component. A fraction of miRNAs are released from living cells into the extracellular environment via the following mechanisms: (1) sorting into multivesicular bodies (MVB) and secretion via exosomes, (2) incorporating into microvesicles that are formed by the outward shedding of the plasma membrane, (3) associating with RNA-binding proteins, such as AGO2 and release of the miRNA-AGO complexes, and (4) exporting and incorporating into high-density lipoprotein (HDL) particles. Extracellular vesicle miRNAs are possibly involved in cell-cell communication.

Intercellular miRNAs have important functions in many biological processes. Current studies in the field of lung disease have shown that miRNAs are present in extracellular spaces, such as blood, urine, and bronchoalveolar lavage fluid (BALF) [28]. Mitchell et al. firstly provided the direct evidence that circulating miRNAs derived from human prostate cancer xenografts are released into mouse circulation [23]. The release mechanism of circulating miRNAs as an active biological process remains largely unclear. The secretion of miRNAs by cells is related to the microenvironment of cells. Some possible mechanisms have been hypothesized for miRNA release: (1) active secretion of miRNAs in the form of vesicle-containing miRNAs and (2) energy-free passive leakage of cellular miRNAs from broken cells [29]. In fact, circulating miRNAs have been found to be packaged into various membrane-bound vesicles, such as exosomes and microvesicles [26] and to exist in a vesicle-free form associated with protein or high-density lipoprotein complexes [24] (Figure 1). Furthermore, miRNAs can be incorporated into apoptotic bodies [30]. Although the terms* exosomes* and* microvesicles* frequently are used interchangeably, these vesicles differ in their sources and the mechanisms of derivation and have distinct structural and biochemical properties that are likely to affect their roles [31]. Some methods for isolating circulating miRNAs from exosomes and microvesicles in human plasma have been developed, such as ultracentrifugation [32] and the ExoQuick precipitation method [33]. However, the exosomal miRNA profiles are affected by the different extracellular vesicle isolation methods [34]. In addition, it is technically difficult to fully discriminate between exosomes and microvesicles using these collection methods. Therefore, we will use the term extracellular vesicle (EV) in this review, according to the definition of the International Society for Extracellular Vesicles (ISEV), when describing studies using ultracentrifugation to isolate EVs [35]. EVs, especially exosomes, are small membrane vesicles that are released extracellularly after the fusion of multivesicular bodies (MVB) with the cell membrane [27]. On the other hand, microvesicles are vesicles shed by the plasma membrane of healthy cells [36]. The difference between these two terms is based on size of the vesicles: exosomes are in the range of 10–100 nm and microvesicles are in the range of 100–1000 nm [37]. Importantly, it has been suggested that EVs carry communications between cells, allowing for cells to promote biological functions at distant sites [38]. However, they may not be the most prevalent form of circulating miRNAs. Arroyo et al. showed that the majority of serum miRNAs are present as AGO2-miRNA complexes but not within EVs [24]. Significantly, only EV-miRNAs reportedly have a function in communicating between cells and play a role in various biological processes, including immune system regulation, inflammation, and tumor development [28, 31]. Therefore, we consider that EV-miRNAs make it possible to reflect every aspect of human physiological status and provide the advantage of being better biomarkers than other circulating miRNAs.

## 3. The Potential of MicroRNAs as Circulating Biomarkers in Cancer Patients

Early cancer detection and improved therapeutic response prediction remain the major challenges in cancer research. Currently, tumor cells have been demonstrated to secrete miRNAs into body fluids [2]. With the development of detection technologies, including RT-PCR, microarray, and deep sequencing, we can screen for circulating miRNAs and generate miRNA signatures in body fluids. Circulating miRNAs are novel minimally invasive diagnostic tools for the detection and risk assessment of various types of cancer. Notably, circulating miRNAs levels in serum or plasma could correlate with cancer progression, therapeutic responses, and survival [39]. In general, miRNAs remain stable in the blood circulation. This high stability is due to their resistance to RNase activity, temperatures, extremes of pH, and extended storage in frozen conditions [40]. This evidence suggests that miRNAs could also be used for diagnostic cancer screening or as noninvasive biomarkers for disease monitoring [41]. Blood-based tests would be more reasonable, as they have relatively low cost and can be repeated as well.

Several researches have revealed that circulating miRNA levels are higher in cancer patients than in healthy donors. Tumor-specific miRNAs (miR-21) were first reported in the serum of patients with diffuse large B-cell lymphoma (DLBCL), indicating that circulating miRNAs can be used as biomarkers to monitor the existence of cancer cells [42]. After this research, many studies have analyzed the clinical relevance of circulating miRNAs in blood for diagnosis and survival [23, 43, 44]. To date, differential expression of circulating miRNAs has been reported in cancers of the lung [44], breast [43], liver [45], kidney [46], bladder [47], and prostate [23], among others. Particularly, Mitchell et al. clearly showed that circulating miRNAs originate from tumors and are protected from endogenous RNase activity, suggesting the high potential for using circulating miRNAs as blood biomarkers for cancer [23]. Their most important advantage is the possibility for repeated measurements in a noninvasive manner. Easy access and superior stability in blood plasma and serum also add to their potential as cancer biomarkers [24, 48]. Remarkably, highly expressed circulating miRNAs from cancer patients have been reported to return to a normal level after tumor resection. Yamamoto et al. have reported that high levels of miR-500 were found in the serum of patients with hepatocellular carcinoma, while the circulating miR-500 returned to normal levels after surgical operation in three of the patients [49]. Serum levels of upregulated miRNAs such as miR-195 and* let-7a* are significantly higher in preoperative plasma from breast cancer patients than after resection [50]. Yamada et al. have reported that surgical removal of urothelial carcinoma coincided with a reduction in urine miR-96 and miR-183 levels [51]. These findings have supported the idea that the level of circulating miRNAs may reflect the expression level of tumor miRNAs.

## 4. Circulating MicroRNAs in Lung Cancer Patients

Lung cancer is often diagnosed in the late stages with poor prognoses. Therefore, it is important to develop biomarkers to identify early-stage lung cancer and to predict therapeutic responses to chemotherapy or radiotherapy. Circulating miRNAs have been proposed as attractive candidates to be used as cancer biomarkers and are ideal for monitoring by screening. So far, several studies have analyzed the potential diagnostic and prognostic role of circulating miRNAs (Table 1).

Chen et al. used deep sequencing followed by qRT-PCR to identify two NSCLC-specific serum miRNAs (miR-25 and miR-223) [44]. This is the first comprehensive analysis of miRNA profiles in the serum of patients with NSCLC using Solexa sequencing technology. After this study, a lot of research aimed to identify circulating miRNAs with a diagnostic relevance in body fluids such as serum, plasma, sputum, and pleural effusion [44, 52–57]. Bianchi et al. evaluated a panel of 361 miRNAs in patients from the COSMOS trial to focus on the diagnostic accuracy of circulating miRNAs in NSCLC [52]. This study reports a combination of two differentially expressed miR-15b and miR-27b capable of discriminating NSCLC from healthy donors with high sensitivity and specificity in the training set. This suggests that serum miRNAs have the promising potential to be sensitive and cost-effective biomarkers for the early detection of NSCLC. Although various miRNAs were identified as biomarkers capable of distinguishing between NSCLC patients and healthy donors, different research identified different promising miRNAs.

Recently, several groups have revealed defined serum or plasma miRNA signatures with prognostic and predictive clinical relevance [41, 58, 59]. The most comprehensive study for circulating miRNAs related to prognosis has been conducted by Hu et al. [41]. In a follow-up validation set of 243 NSCLC patients, the serum levels of four miRNAs (miR-486, miR-30d, miR-1, and miR-499) were significantly related to a poor prognosis. When these miRNAs were combined to generate the four miRNA signatures, patients with high-risk signatures had a 9.31-fold increased hazard ratio for cancer death and a shorter median survival. Following computed tomography screening, Boeri et al. showed that 9 circulating miRNAs (miR-221, miR-660, miR-486-5p, miR28-3p, miR-197, miR-106a, miR-451, miR-140-5p, and miR-16) were found to indicate a risk of lung cancer malignancy and poor prognosis [58]. These data suggest that circulating miRNAs can be released into the extracellular spaces even several years before the clinical manifestation of lung cancer. Recently, a significant correlation was reported between serum miR-21 expression and clinicopathological factors of NSCLC patients [60]. High serum levels of this miRNA were significantly related to lymph node metastasis, advanced clinical staging, and a poor prognosis. Moreover, vesicle-related miRNAs have also been quantified and found to be significant cancer prognosis biomarkers (*let-7f* and miR-30e-3p) [53].

In advanced NSCLC patients receiving cisplatin-based chemotherapy, high expression level of circulating miR-125b was a diagnostic and prognostic marker [61]. In addition, the high serum level of miR-125b was shown to be associated with poor sensitivity to neoadjuvant chemotherapy in various types of cancer [61, 62]. This data strongly implied the feasibility of miRNAs as noninvasive or circulating biomarkers for not only the early detection of lung cancer but also its chemoresistance.

Despite promising developments in this research field, circulating miRNAs as biomarkers for cancer need to be sufficiently investigated to validate their potential. Actually, it is unclear from which types of cells the diagnostically relevant miRNAs are derived. It cannot be excluded that circulating miRNAs might be derived from a variety of cell types, such as blood cells, tumor-associated fibroblasts, endothelial cells, and various immune cells residing in the tumor microenvironment. Therefore, we must analyze the miRNA profiles expressed by noncancerous cells, which could reflect specific inflammatory or immune-modulatory processes that occur during carcinogenesis.

## 5. Extracellular Vesicle-Encapsulated Circulating MicroRNAs in Cancer Progression

EV miRNAs are derived from normal and tumor cells in body fluids as an active secretion mechanism and can mediate cell-cell communication. EVs containing mRNA, miRNAs, and angiogenic proteins released by cancer cells have been revealed [63]. The Swedish group first showed the remarkable discovery that miRNAs are contained inside EVs, which are lipoprotein complexes, including small membrane vesicles of endocytic origin [26]. They demonstrated that mouse and human mast cell-derived EVs contain RNA and miRNA and that RNA from mast cell EVs is transferable to other mouse and human mast cells. In 2010, three groups reported that EV miRNAs are transferred to recipient cells and suppress the expression of target genes [64–66]. Over the last decade, EVs have become the next focus of intensive scientific research as novel mediators of intercellular communication. Recent studies have shown the deep involvement of tumor cell-derived EVs in cancer progression. Moreover, EVs secreted from the surrounding cells have also been reported to regulate tumor pathology [67]. Kosaka et al. reported that EV miRNA-210 released by metastatic breast cancer cells entered endothelial cells and suppressed the expression of its target genes, which resulted in enhanced angiogenesis [68]. Rana et al. reported that EV miR-494 and miR-542-3p derived from rat adenocarcinoma cells modulate premetastatic organ cells predominantly through transferred miRNA, where miRNA from a metastasizing tumor prepares premetastatic organ stroma cells for tumor cell hosting [69]. Fabbri et al. found that EV miR-21 and miR-29a can induce a Toll-like receptor (TLR)- mediated prometastatic inflammatory response by binding to TLRs regulating for the tumor microenvironment [70]. Recently, Zhou et al. found that EV miR-105 can destroy vascular endothelial barriers to promote brain metastasis [71]. These data suggest the deep involvement of cancer cell-derived EVs in cancer malignancy such as metastasis. Therefore, EV miRNAs have potential not only to be putative biomarkers but also to reflect cancer progression. For this reason, we suggest that EV miRNAs may have the potential of prognostic biomarkers to predict clinical outcome of lung cancer patients. So far, there are few reports that vesicle-free forms associated with protein or HDL complexes can mediate the expansion of cancer malignancy in an autocrine or paracrine manner [25]. The functionality of EV miRNAs in the recipient cells has opened up a new avenue of cell-cell communication in cancer biology, including lung cancer research.

## 6. Conclusions

**Figure 2:**
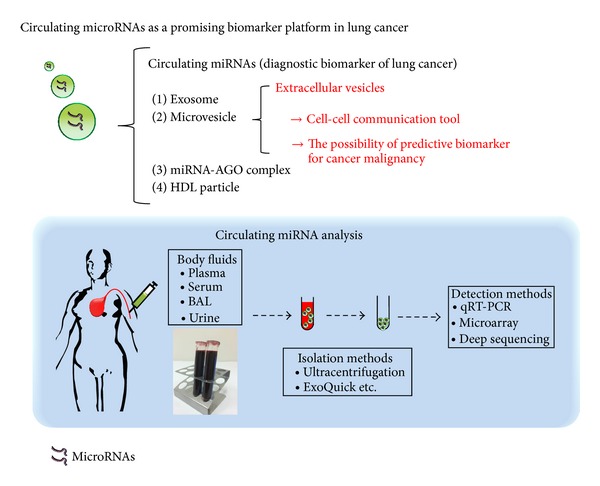
Circulating microRNAs as a promising biomarker platform in lung cancer. Circulating miRNAs have been found to be packaged into various membrane-bound vesicles, such as exosomes and microvesicles and to exist in a vesicle-free form associated with protein or high-density lipoprotein complexes. Extracellular vesicles (EVs), such as exosomes and microvesicles, are recognized as novel tools for cell-cell communication and might be predictive biomarkers for cancer malignancy. EVs that circulate in body fluids can be readily recovered using several existing isolation methods and analyzed by various detection methods.

Circulating miRNAs are promising lung cancer biomarker candidates (Figure 2). Specific tumor-associated circulating miRNA signatures will have to be developed as early biomarkers for lung cancer. Remarkably, some studies have succeeded in discovering circulating miRNAs with prognostic or predictive significance. In addition, it was reported that some circulating miRNAs are strongly associated with distant metastasis, disease stage, and survival. Regarding this point, we consider that only EV miRNAs may contribute to the management of cancer progression. Therefore, EV miRNAs will be suitable predictive biomarkers for cancer malignancy metastasis and prognosis. We emphasize that it is important to investigate the role of EV miRNAs in cell-cell communication and to explore the effectiveness of these molecules as biomarkers in lung cancer research. As we have shown, circulating miRNAs have been found to be packaged into various membrane-bound vesicles, such as exosomes, microvesicles, and protein complexes. It is technically difficult to completely discriminate between these vesicles using simple collection methods; therefore, we need to collect vesicles using ultracentrifugation when we research the biology of EVs. Gaining deeper understanding of the methods of circulating miRNA detection and analysis will help to clarify the information carried by circulating miRNAs and lead to novel therapeutic approaches targeting the circulating miRNAs.

## Figures and Tables

**Table 1:** Circulating miRNAs as a potential biomarker for lung cancer.

Study design	Body fluid type	MicroRNA	Detection method	Reference
NSCLC versus normal	Serum	miR-25 and -223	NGS, qRT-PCR	[44]
NSCLC versus normal	Serum	miR-15b and -27b	qRT-PCR	[52]
NSCLC versus normal	Plasma vesicles	*let-7f* and miR-30e-3p	qRT-PCR array	[53]
Stage I/II versus IV NSCLC	Serum	miR-126 and -183	qRT-PCR	[54]
NSCLC versus normal	Plasma	miR-21, -126, -210, -486-5p	qRT-PCR	[55]
NSCLC versus normal	Sputum	miR-31, -210	qRT-PCR	[56]
NSCLC versus normal	Sputum	miR-31, -210	qRT-PCR	[57]
NSCLC versus normal (metastasis stage)	Serum	miR-21	qRT-PCR	[60]
Prognosis of NSCLC	Serum	miR-486, -30d, -1, -499	NGS, qRT-PCR	[41]
Prognosis of NSCLC	Plasma	miR-221, -660, -486-5p, -28-3p, -197, -106a, -451, -140-5p, -16	qRT-PCR array	[58]
Prognosis of NSCLC	Serum	miR-21, -141, -200c	qRT-PCR	[59]
Response parameter for chemotherapy	Serum	miR-125b	qRT-PCR	[61]

NGS: next generation Solexa sequencing; qRT-PCR: quantitative real-time polymerase chain reaction; miR: microRNA; NSCLC: non-small-cell lung cancer.
